# Prediction of COVID-19 transmission dynamics using a mathematical model considering behavior changes in Korea

**DOI:** 10.4178/epih.e2020026

**Published:** 2020-04-13

**Authors:** Soyoung Kim, Yu Bin Seo, Eunok Jung

**Affiliations:** 1 Department of Mathematics, Konkuk University, Seoul, Korea; 2 Division of Infectious Diseases, Department of Internal Medicine, Hallym University College of Medicine, Chuncheon, Korea

**Keywords:** COVID-19, Mathematical model, Behavior changes, Parameter estimation, Model prediction

## Abstract

**OBJECTIVES:**

Since the report of the first confirmed case in Daegu on February 18, 2020, local transmission of coronavirus disease 2019 (COVID-19) in Korea has continued. In this study, we aimed to identify the pattern of local transmission of COVID-19 using mathematical modeling and predict the epidemic size and the timing of the end of the spread.

**METHODS:**

We modeled the COVID-19 outbreak in Korea by applying a mathematical model of transmission that factors in behavioral changes. We used the Korea Centers for Disease Control and Prevention data of daily confirmed cases in the country to estimate the nationwide and Daegu/Gyeongbuk area-specific transmission rates as well as behavioral change parameters using a least-squares method.

**RESULTS:**

The number of transmissions per infected patient was estimated to be about 10 times higher in the Daegu/Gyeongbuk area than the average of nationwide. Using these estimated parameters, our models predicts that about 13,800 cases will occur nationwide and 11,400 cases in the Daegu/Gyeongbuk area until mid-June.

**CONCLUSIONS:**

We mathematically demonstrate that the relatively high per-capita rate of transmission and the low rate of changes in behavior have caused a large-scale transmission of COVID-19 in the Daegu/Gyeongbuk area in Korea. Since the outbreak is expected to continue until May, non-pharmaceutical interventions that can be sustained over the long term are required.

## INTRODUCTION

In December 2019, a case of pneumonia of unknown cause occurred in Wuhan, China and was identified as a novel coronavirus infection in January 2020. Originating from Wuhan, the virus then spread to every region in China. Since the report of the first confirmed case outside China in Thailand on January 13, 2020, the virus has been spreading worldwide. On January 30, 2020, the World Health Organization (WHO) declared the outbreak a public health emergency of international concern [1]. According to the WHO situation reports No. 48 [2], a total of 53 countries so far, including Korea, Italy, and Japan are showing community transmission; there have been 80,859 confirmed cases in China and 24,727 confirmed cases outside of China as of March 8, 2020.

**Figure 1.**
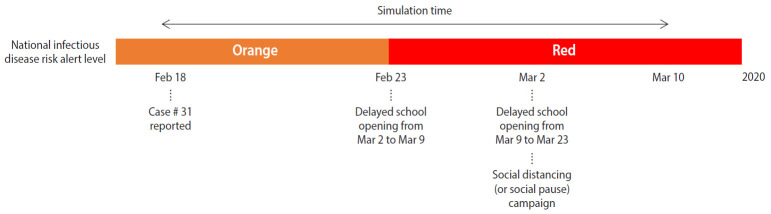
Timeline of coronavirus disease 2019 epidemic.

In Korea, after the first confirmed case of coronavirus disease 2019 (COVID-19) on January 20, 2020, the epidemic has continued to spread. Prior to February 18, 2020, most confirmed cases were imported or a result of transmission via close contact with a confirmed patient, but since February 18, 2020, local transmission has led to a spread throughout the entire nation following outbreaks in religious communities and social welfare facilities. On February 23, 2020, the Korea Centers for Disease Control and Prevention (KCDC) raised the infectious disease alert level to “red,” the highest level, and declared that it would implement a containment policy including patient detection and isolation along with strategies to prevent and minimize local transmission [3]. The government is actively intervening by tracking and controlling those who have been in close contact and conducting disease prevention activities, while the public is making efforts to prevent the transmission through social distancing and improvements in personal hygiene. Figure 1 shows the infectious disease alert level and non-pharmaceutical interventions set in place by the government from February 18, 2020 to March 10, 2020.

We applied a mathematical model that factors in the behavioral changes that were implemented in the population since local transmission began on February 18, 2020, to estimate the transmission rate. The model predicts the final size and the timing of the end of the epidemic as well as the maximum number of isolated individuals. While the number of confirmed cases continues to increase nationwide, the number of cases in the Daegu/Gyeongbuk area accounts for about 90% of all cases in Korea, and the incidence rate per 100,000 population in the Daegu/Gyeongbuk area, at 132.96 cases on average (Daegu, 232.42; Gyeongbuk, 41.95), is at least nine times higher than the national average of 14.49 (as of March 10, 2020) [4]. Our mathematical transmission model, therefore, discriminates between the national level and the Daegu/Gyeongbuk area. We, thus, analyzed the pattern of COVID-19 local transmission in Korea by mathematical modeling and simulations and estimated the relative risk by comparing epidemiological parameters between the national level and the Daegu/Gyeongbuk area.

## MATERIALS AND METHODS

### Data

We used the data from the KCDC daily press releases [5] and the daily confirmed cases from February 18, 2020, when the case #31 was confirmed, to March 10, 2020. The daily case data from February 18, 2020 to March 1, 2020 were updated at 9:00 a.m., while from March 2, 2020, data were updated at 0:00 a.m. We calculated the number of cases by region based on the initially reported cases. As an estimate of the total population, we used the resident registration data as of February 2020, provided by the Korean Statistical Information Service [6].

### Mathematical model

**Figure 2.**
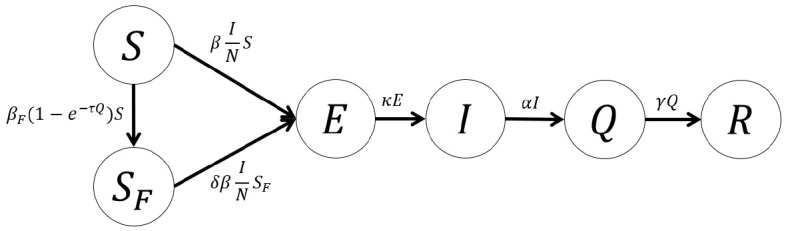
Flow diagram of coronavirus disease 2019 transmission dynamics. *S*, consists of susceptible; *S_F_*, behavior-changed susceptible; *N*, total population; *E*, virus-exposed; *I*, infectious; *Q*, hospital-quarantined; *R*, recovered.

We constructed a mathematical model of COVID-19 transmission based on the SEIR model with a hospital-quarantined group. A behavior-changed group, strives to reduce the transmission rate by social distancing and other measures, is also considered in this work. For the behavioral changes of the susceptible group, we modified the model of prevalence-based behavioral change proposed by Perra et al. [7] (global, prevalencebased spread of the fear of the disease) to fit the situation in Korea. The total population (*N*) consists of susceptible (*S*), behavior-changed susceptible (*S_F_*), virus-exposed (*E*), infectious (*I*), hospital-quarantined (*Q*), and recovered (*R*) individuals. Figure 2 shows the flow diagram of COVID-19 transmission according to our model, and the transmission dynamics can be described with the following differential equations.

$$\frac{dS}{dt}=-\beta \frac{I}{N}S-\beta_{F}(1-e^{-\tau Q})S,\;\frac{dS_{F}}{dt}=\beta_{F}(1-e^{-\tau Q})S-\delta \beta \frac{I}{N}S_{F},\;\frac{dE}{dt}=\beta \frac{I}{N}S+\delta \beta \frac{I}{N}S_{F}-\kappa E,\;\frac{dI}{dt}=\kappa E-\alpha I,\;\frac{dQ}{dt}=\alpha I-\gamma Q,\;\frac{dR}{dt}=\gamma Q,\;N=S+S_{F}+E+I+Q+R.$$

As the number of confirmed cases increases, the susceptible population moves to the behavior-changed susceptible group as people strive to improve personal hygiene, for example by wearing a mask, and practice social distancing, for example by refraining from mass gatherings or meetings, in awareness or fear of the spread of the virus. Both the normal susceptible group (*S*) that did not implement any changes in behavior as well as the behavior-changed susceptible group (*S_F_*) can be exposed to the virus via contact with infected patients, but we assumed that the probability of transmission is decreased in the behavior-changed susceptible group.

Individuals who were exposed to the virus via contact with infectious patients move to the virus-exposed group (*E*) and develop symptoms (*I*) after the virus incubation period. After symptoms onset, infectious individuals (*I*) visit the hospital, become confirmed and quarantined (*Q*). Finally, they move to the recovered group (*R*) after a recovery period. In this study, we assumed that individuals are hospital quarantined after a confirmation and, thus, cannot infect others.

We set the incubation period of the virus to 4.1 days, referring to the KCDC regular briefing on February 16, 2020 [8], and the mean period from symptom onset to confirm and isolation to 4 days [9]. Period from isolation to recovery was set to 14 days, which was the mean of 16 discharged patients according to the detailed information on confirmed cases reported until February 19, 2020. Mortality was not included in the present model as it was difficult to calculate the exact value at this point. Table 1 shows the description and values of the parameters used in the model. To define the initial value of the model, we used the #31 case confirmed on February 18, 2020 as *Q*(0).

### Ethics statement

This research is based on data which is open to public. Neither ethical approval of an institutional review board nor written informed consent we required.

## RESULTS

**Figure 3.**
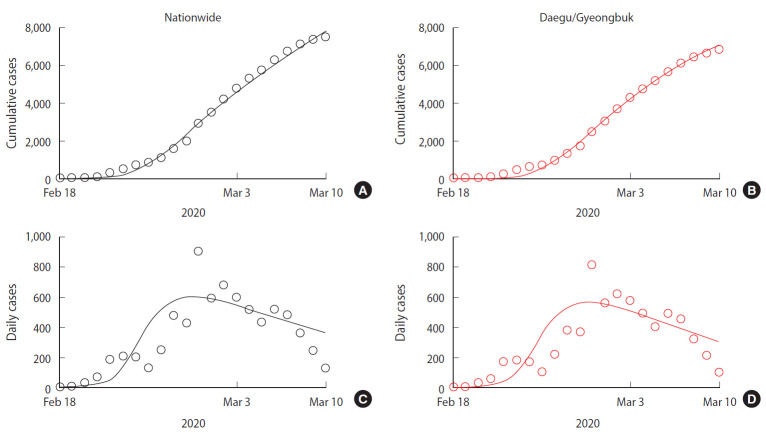
Data fittine results (A) cumulative cases in nationwide, (B) daily cases in nationwide, (C) cumulative cases in Daegu/Gyeongbuk province, and (D) daily cases in Daegu/Gyeongbuk province. Confirmed cases data (circle) from February 18, 2020 to March 10, 2020 together with the model estimation (solid curve).

To estimate the transmission rate and the rate of individuals moving from the susceptible to the behavior-changed susceptible group, we used a least-squares fitting method that minimizes the sum of the squares of the differences between the cumulative cases data and the model curve. The estimated values for all parameters, for the Daegu/Gyeongbuk area and nationwide, are presented in Table 1. Figure 3 shows the nationwide (A and C) and Daegu/Gyeongbuk (B and D) confirmed cases data (circle) from February 18, 2020 to March 10, 2020 together with the model estimation (solid curve). Figure 3A and 3B shows the number of cumulative confirmed cases and Figure 3C and 3D shows the number of new confirmed cases per day.

**Figure 4.**
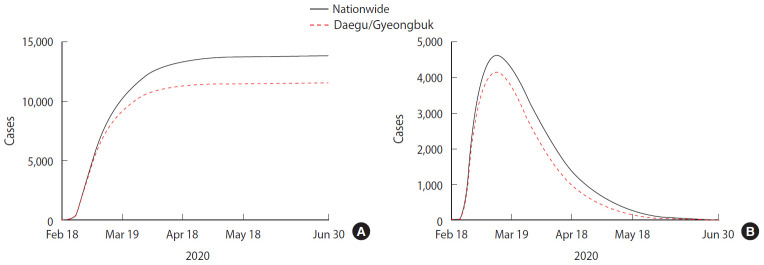
Model prediction (A) cumulative confirmed cases and (B) isolated cases using estimated parameters.

We predicted the timing of the end of the COVID-19 outbreak and the total number of confirmed patients using the estimated parameters described above and obtained the following results. The end point of the COVID-19 outbreak is defined as a date that the number of expected daily new case is less than 1. Figure 4A shows the number of cumulative confirmed cases over time, while Figure 4B shows the number of isolated patients over time. The predicted values of nationwide cases are represented by the black solid line and the Daegu/Gyeongbuk cases by the red dashed line.

Table 2 summarizes model predictions. According to the model, approximately 13,800 cases are expected to occur nationwide, and the date of the last confirmed case will be June 14, 2020. In the Daegu/Gyeongbuk region, approximately 11,400 cases are expected to occur until May 27, 2020. The proportion of Daegu/Gyeongbuk cases by the end of the outbreak is estimated at 82.7%. The number of cumulative cases per 100,000 people is predicted to be 26.49 nationwide and 224.64 in the Daegu/Gyeongbuk area. The number of isolated patients, both nationwide and in Daegu/Gyeongbuk, will peak on March 11, 2020, and then start to decrease. The maximum number of isolated individuals predicted by the model is 4,625 nationwide, of which 4,167 are predicted to be located in the Daegu/Gyeongbuk area.

## DISCUSSION

Assuming that the period from symptom onset to isolation is identical for the cases in the Daegu/Gyeongbuk area and those occurring nationwide, our model estimates the per-capita rate of transmission (*β/N*) to be at least 8.9 times higher in the Daegu/Gyeongbuk area, with 1.3616e-7 per infected patient nationwide and 1.2145e-6 around Daegu/Gyeongbuk. The transmission rate of the behavioral changes implemented in the Daegu/Gyeongbuk area is also estimated to be lower than that of the national average.

In the early stages of the outbreak, the period from symptom onset to isolation was at least 5 days, but recently, most patients are confirmed and isolated within 1-2 days of symptom onset due to the active screening of close contacts by the KCDC. However, as there are still reports of cases of transmission after more than 5 days from symptom onset, we set the mean period from onset to isolation to 4 days in our model. The rate of reduction in transmission in the behavior-changed group significantly affects the model results. Since the rate of reduction in transmission that results from the currently implemented social distancing policies has never been studied, we factored in the number of people working at home and the delayed start of classes and concluded that the transmission rate will decrease by about 1/50 or more. In our model, a decrease in the parameter, which means transmission rate of behavior-changed susceptible is decreased more, results in a decrease in the total number of confirmed cases and the duration of the outbreak.

In the present study, we assumed that the susceptible group that implemented behavioral changes maintains a low transmission rate. However, when schools open again after March 24, 2020, an increase in the number of contacts and a desensitization of the behavior-changed group with a decreasing number of patients may lead to another cluster outbreak stemming from unconfirmed positive cases.

Since this model assumes that patients are isolated at diagnosis and cannot spread the virus further, the recovery period and mortality rate do not affect the total number of patients, but they only affect the calculation of the number of isolated patients and the required inpatient beds. The number of daily isolated patients increases with an increasing recovery period and decreases with an increasing mortality rate.

The COVID-19 pandemic is characterized by large-scale cluster outbreaks in specific areas. A large-scale outbreak within a region beyond the capacity of local hospitals can lead to some confirmed patients having to self-quarantine, during which they might infect other people. It is, thus, necessary to predict the number of isolated patients over time through modeling to identify the number of beds required in advance and establish a response strategy.

While in Korea, a large-scale outbreak occurred in the Daegu/Gyeongbuk area, the results of our mathematical model show that up to about 1,000 confirmed cases may occur in regions outside Daegu/Gyeongbuk as well. As part of the government’s active intervention policy, powerful non-pharmaceutical interventions such as postponing the start of classes in elementary/middle/high schools and reducing mass gatherings are being implemented. However, given that the outbreak is expected to continue through June 2020, it will be difficult to sustain the strengthened nonpharmaceutical interventions. Therefore, even after schools open again, it will be necessary to suggest sustainable non-pharmaceutical intervention guidelines, for example reinforcing personal hygiene practices such as mask wearing and hand washing, encouraging people to avoid close contact and visit a medical center quickly after suspicious symptoms develop, and shortening the time from symptom onset to isolation.

## Figures and Tables

**Table 1.** Parameter description and values

Symbol	Description	Value		Reference
		Nationwide	Daegu/Gyeongbuk	
*β*	Transmission rate of coronavirus disease 2019 disease	7.059	6.184	Data-fitted
*β_F_*	Transmission rate of the awareness/fear of the disease	4.811	4.085	Data-fitted
1/τ	Characteristic no. of confirmed individuals reported in the news		1,000	Assumed
*δ*	Transmission reduction ratio of behavior-changed individuals		0.02	Assumed
*κ*	Progression rate		1/4.1	[8]
*α*	Isolation rate		1/4	[9]
γ	Recovery rate		1/14	[5]

**Table 2.** Model predictions

Region	Maximum no. of isolated individuals	Peak of isolated individuals	Final size of epidemic^1^	Final incidence rate per 100,000	End of outbreak
Nationwide	4,625	Mar 11, 2020	13,830	26.49	Jun 14, 2020
Daegu/Gyeongbuk	4,167	Mar 11, 2020	11,438	224.64	May 27, 2020

1 Except 30 cases before February 18, 2020.
