# Supplementary material for: Prediction of COVID-19 transmission dynamics using a mathematical model considering behavior changes in Korea
Source: Epidemiol Health. 2020 Apr 13;42:e2020026. doi: 10.4178/epih.e2020026 (PMC7285444; doi:10.4178/epih.e2020026)
Supplement: Supplementary file 1 [file epih-42-e2020026-suppl.pdf]

행동변화를 고려한 코로나바이러스감염증-19 수리모델링 및 감염 전파 양상 예측

Prediction of COVID19 transmission dynamics using mathematical model considering behavior changes

Abstract

목적(Objectives): 2020년 2월 18일 대구에서 첫 환자가 보고된 이후 코로나바이러스감염증-19의 지역사회 감염이 계속되고 있다. 본 연구에서는 수학적 모델링을 이용하여 코로나바이러스감염증-19의 지역사회 전파 양상을 파악하고 확산 규모와 종료 시점을 예측하고자 한다.

방법(Methods): 행동변화를 고려한 감염전파 수학적 모델을 적용하여 우리나라 코로나바이러스감염증-19유행을 모델링하였다. 질병관리본부의 일별 코로나 확진자 국내현황 데이터를 사용하여 전국과 대구·경북지역의 감염전파율과 행동변화 모수를 최소제곱법으로 추정하였다.

결과(Results): 감염환자 1명당 감염전파 환자수가 대구·경북지역에서 약 10배정도 높은 것으로 계산되었다. 추정된 모수를 이용하여 총 유행 규모와 종료시점을 예측한 결과 전국적으로 6월 중순까지 약 13,800명 대구경북지역의 경우 약 11,400명의 환자가 발생할 것으로 예상된다.

결론(Conclusion): 상대적으로 높은 인구당 감염전파율과 낮은 행동변화율로 인해 대구·경북지역의 코로나바이러스감염증-19 대규모 전파확산을 초래하였음을 수치적으로 확인하였다. 5월까지 환자 발생이 지속적으로 발생할 것으로 예측됨에 따라 장기적으로 지속 가능한 비약물적 중재가 필요하다.

중심단어(Key words)

코로나바이러스감염증-19(COVID19), 수학적 모델링(Mathematical modeling), 행동변화(Behavior change), 모수 추정(Parameter estimation), 모델 예측(Model prediction)

List of Abbreviation

S: Susceptible

S_F: Behavior changed susceptible

E: Exposed

I: Infectious

Q: Isolated

R: Recovered

KCDC: Korea Center for Disease Control and Prevention

WHO: World Health Organization

Introduction

2019년 12월 중국 우한시에서 원인 불명의 폐렴 환자가 발생하였고 2020년 1월 이는 새로운 코로나바이러스에 인한 환자 발생임이 파악되었다. 이 바이러스는 중국 우한시를 거점으로 중국 전역으로 전파되었다. 1월 13일 중국 외의 국가에서 처음으로 환자가 보고된 이후 전세계적으로 바이러스가 전파됨에 따라 세계보건기구(World Health Organization, WHO)에서는 1월 30일 국제적 공중보건 비상사태(Public health emergency of international concern)를 선포하였다(1). WHO 상황보고서에 따르면 현재까지 한국, 이탈리아, 일본을 비롯하여 총 53개국에서 지역사회 감염이 진행되고 있으며 중국 내 80,859명, 이외국가에서 24,727명이 발생하였다 (2020년 3월 8일 기준)(2).

우리나라의 경우 2020년 1월 20일 코로나바이러스감염증-19 첫 환자 발생 이후 3월 9일 현재까지 유행이 지속되고 있다. 2월 18일 이전까지 대부분의 확진환자가 해외에서 유입되거나 확진환자의 밀접접촉자 중심으로 유행이 전파된 것에 반해 2월 18일 이후 확진자의 경우는 종교시설, 사회복지시설 등에서의 집단발생 중심으로 지역사회 전파가 전국으로 확산되었다. 질병관리본부에서는 2월 23일부터 감염병 위기경보를 최고단계인 심각으로 격상하면서 환자 발견 및 접촉자 격리 등의 봉쇄 정책과 더불어 지역사회 확산 차단 및 최소화 전략을 병행한다고 선언했다(3). 정부 차원의 적극적인 중재정책으로 밀접접촉자 추적 및 관리와 방역활동을 진행하고 있고, 국민들은 사회적 거리두기(social distancing), 개인위생제고 등을 통하여 감염전파 차단을 위해 노력하고 있다.

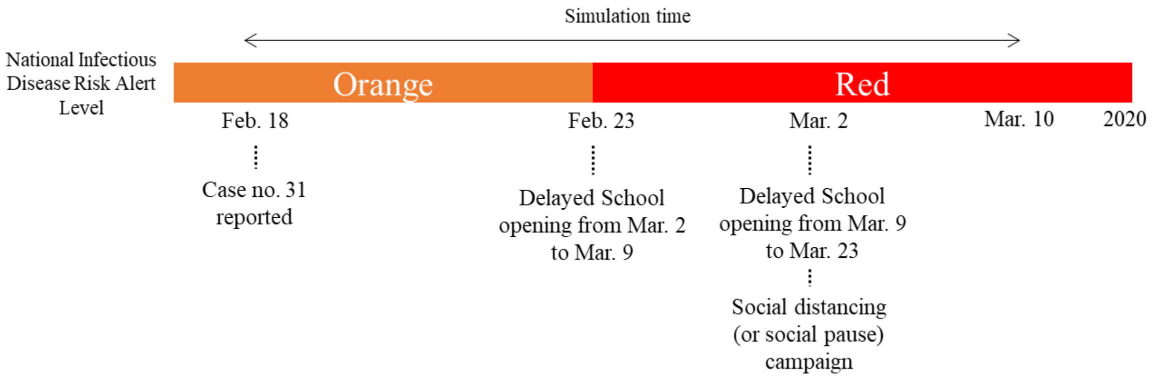

Figure **1**는 2월 18일부터 3월 10일까지의 감염병 위기경보 단계와 정부의 비약물적 중재전략을 나타낸다.

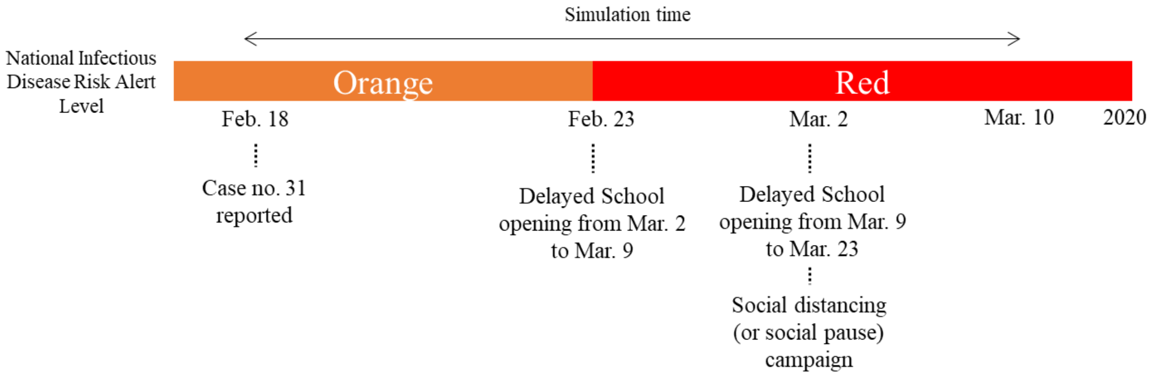

Figure 1 Timeline of COVID-19 epidemic

본 연구에서는 지역사회 감염이 본격적으로 시작된 2월 18일부터 행동변화를 고려한 수리모델을 적용하여 감염전파율을 추정하고, 유행의 규모, 종료시점, 최대 격리자수를 예측하였다. 전국에서 지속적으로 환자가 보고되고 있지만 대구·경북 지역의 환자수가 전국환자수의 약 90%을 차지하고 있으며 인구 10만명당 발생률도 대구·경북지역 평균 132.96명(대구 232.42, 경북 41.95)으로 전국평균인 14.49보다 9배 이상 높게 보고되었다 (2020년 3월 10일 기준)(4). 따라서 본 연구에서는 전국단위의 코로나바이러스감염증-19 감염 전파확산 수리모델링과 대구·경북 지역내에서의 수리모델링을 진행하였다. 수리모델 시뮬레이션 결과 분석을 통해, 한국에서의 코로나바이러스감염증-19 지역사회 감염 전파양상을 파악하고 전국단위와 대구·경북지역에서의 역학적 모수 비교를 통해 상대적 위험도를 산출하였다.

Materials and methods

Data

데이터는 질병관리본부 일일 보도자료(5)를 사용하였으며 31번 환자가 대구에서 확진 판정을 받은 2월 18일부터 3월 10일까지의 일일 확진자 데이터를 사용하였다. 2월 18일부터 3월 1일까지는 09시 기준 일일 확진자 데이터, 3월 2일부터는 0시 기준 일일 확진자 데이터를 사용하였으며 최초 보고된 자료를 기준으로 지역별 환자수를 산정하였다. 전체 인구는 통계청 국가통계포털 2020년 2월 주민등록인구를 사용하였다(6).

Mathematical model

코로나바이러스감염증-19의 감염 확산 수리모델링을 위하여 SEIR 모델을 기반으로, 병원 격리 그룹과 사회적 거리두기 등 감염전파율 감소를 위해 노력하는 행동변화 그룹을 고려하여 모델을 구성하였다. 감수성군의 행동변화는 Perra가 발표한 논문에서 유병환자 기준 행동변화 양상(Global, prevalence-based spread of the fear of the disease)을 우리나라 상황에 맞게 변형하여 적용하였다

(7). 전체 인구(N)는 감수성군(S), 행동변화 감수성군(S_F), 바이러스 노출군(E), 감염환자군(I), 병원격리군(Q), 그리고 회복군(R)으로 구성된다. Figure 2은 코로나바이러스감염증-19의 감염전파 흐름도를 나타낸다.

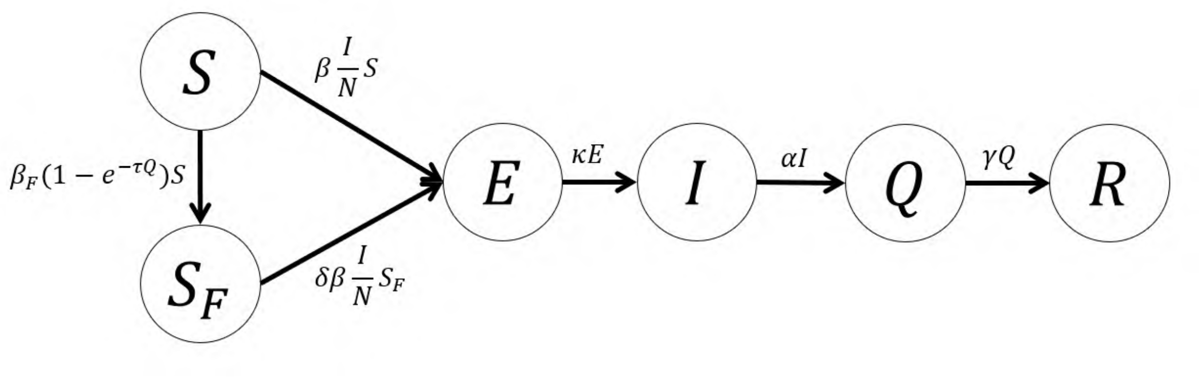

Figure 2 Flow diagram of COVID19 transmission dynamics

본 연구에서는 확진자수가 증가함에 따라서 감수성 인구가 바이러스 전파에 대한 인지 또는 공포심을 가지고 마스크 착용 등의 개인위생 제고, 대규모 집회나 모임 자제 등 사회적 거리두기를 통해 행동 변화 감수성 군으로 이동한다. 행동변화가 진행되지 않은 일반 감수성군(S)과 행동변화가 진행된 감수성군(S_F) 모두 감염환자와의 접촉을 통해 바이러스에 노출될 수 있지만 행동변화 감수성 군의 경우 전파감염확률이 감소한다고 가정하였다. 감염환자와의 접촉을 통해 바이러스에 노출된 사람들은 바이러스 노출군(E)으로 이동하고 바이러스 잠복기간을 거쳐 증상이 발현(I)된다. 증상발현 후 의료기관에 내원하여 확진 및 격리(Q)가 진행되고 회복기간을 거쳐 회복군(R)으로 이동한다. 본 연구에서는 확진과 동시에 병원격리가 되어 더 이상 다른 사람들을 감염시키지 않는다고 가정하였다. 코로나바이러스감염증-19의 감염 전파 수리모델은 다음과 같은 미분방정식으로 나타낼 수 있다.

$$\frac{dS}{dt} = -\beta \frac{I}{N} S - \beta_F(1 - e^{-\tau Q})S,$$
$$\frac{dS_F}{dt} = \beta_F(1 - e^{-\tau Q})S - \delta\beta \frac{I}{N} S_F,$$
$$\frac{dE}{dt} = \beta \frac{I}{N} S + \delta\beta \frac{I}{N} S_F - \kappa E,$$
$$\frac{dI}{dt} = \kappa E - \alpha I,$$
$$\frac{dQ}{dt} = \alpha I - \gamma Q,$$
$$\frac{dR}{dt} = \gamma Q,$$

$$N = S + S_F + E + I + Q + R.$$

바이러스의 잠복기는 질병관리본부 2월 16일 정례브리핑을 참고하여 4.1일(8), 증상발현부터 확진 및 격리까지의 평균 기간은 4일(9)로 설정하였다. 병원격리 후 회복까지의 기간은 2월 19일까지 보고된 확진자 세부데이터를 이용하여 퇴원환자 16명의 평균인 14일로 설정하였다. 사망률의 경우 현재 시점에서 정확한 산출이 어렵다고 판단하여 본 모델에서는 고려하지 않았다. Table 1은 모델에서 사용된 모수의 정의와 값을 나타낸다. 모델의 초깃값은 2월 18일 확진된 31번 확진자를 $Q(0)$로 설정하였다.

Results

감염전파율과 감수성군에서 행동변화 감수성군으로 옮겨가는 비율의 추정은 누적 확진자수 데이터와 모델 결과의 오차의 제곱의 합이 최소가 되게 하는 최소제곱법(least squares fitting) 방법을 사용하였다. 전국과 대구경북 지역에 대한 추정된 이들 모수 값은 Table 1에 있다. Table 1

Parameter description and values

Symbol	Description	Value		Reference
		Nationwide	Daegu, Gyeongbuk	
β	The transmission rate of COVID-19 disease	7.0591	6.1841	Data-fitted
β_F	The transmission rate of the awareness/fear of the disease	4.8106	4.0850	Data-fitted
$1/\tau$	The characteristic number of confirmed individuals reported by the news	1000		Assumed
δ	The transmission reduction ratio of behavioral changed individuals	0.02		Assumed
κ	Progression rate	1/4.1		(8)
α	Isolation rate	1/4		(9)
γ	Recovery rate	1/14		(5)

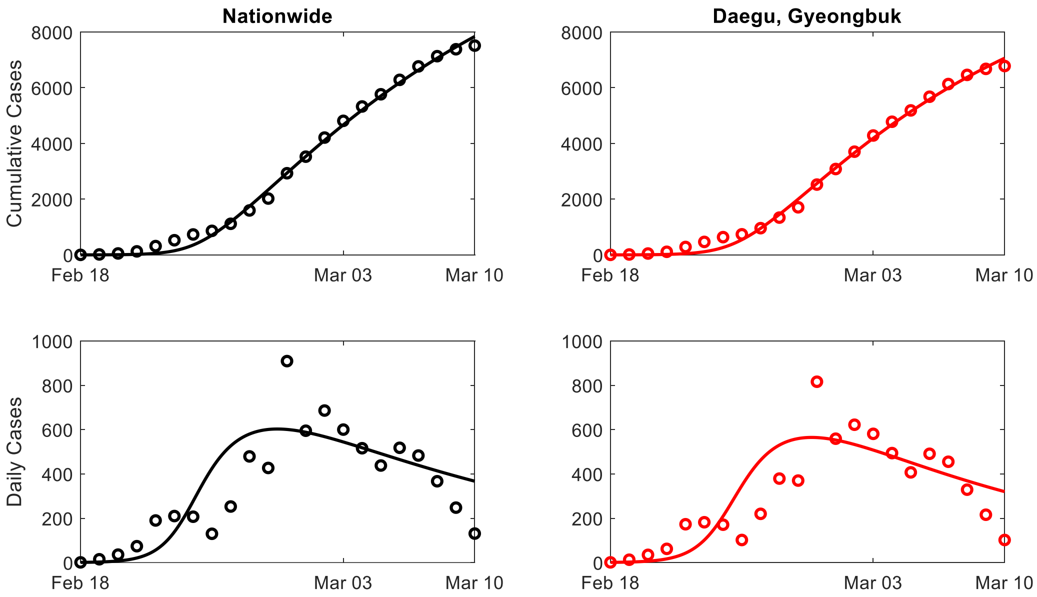

Figure ***3***는 2월 18일부터 3월 10일까지 전국(좌)과 대구·경북(우) 확진자 데이터(동그라미)와 모델 그래프(실선)를 보여준다. 위의 두 그림은 누적 확진자수를 나타내며 아래의 두 그림은 일일 신환자수를 나타낸다.

Table 1 Parameter description and values

Symbol	Description	Value		Reference
		Nationwide	Daegu, Gyeongbuk	
β	The transmission rate of COVID-19 disease	7.0591	6.1841	Data-fitted
β_F	The transmission rate of the awareness/fear of the disease	4.8106	4.0850	Data-fitted
$1/\tau$	The characteristic number of confirmed individuals reported by the news	1000		Assumed
δ	The transmission reduction ratio of behavioral changed individuals	0.02		Assumed
κ	Progression rate	1/4.1		(8)
α	Isolation rate	1/4		(9)
γ	Recovery rate	1/14		(5)

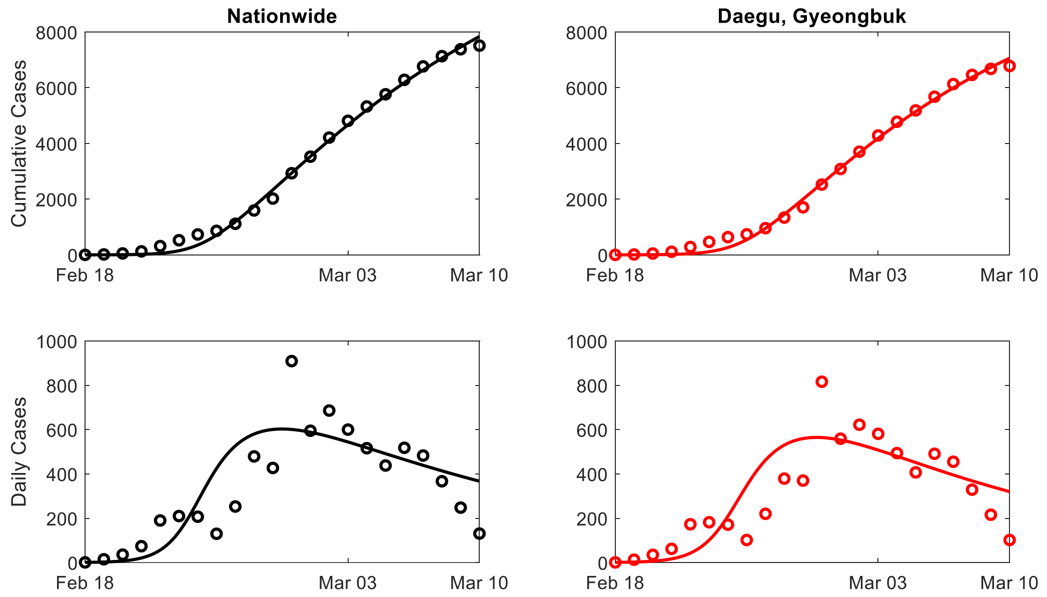

Figure 3 Datafit results

추정된 모수를 이용하여 코로나바이러스감염증-19 유행종료시점과 총 환자수를 예측한 결과는 다음과 같다. 유행종료시점은 일일발생환자 예측치가 1명 미만인 날로 정의하였다.

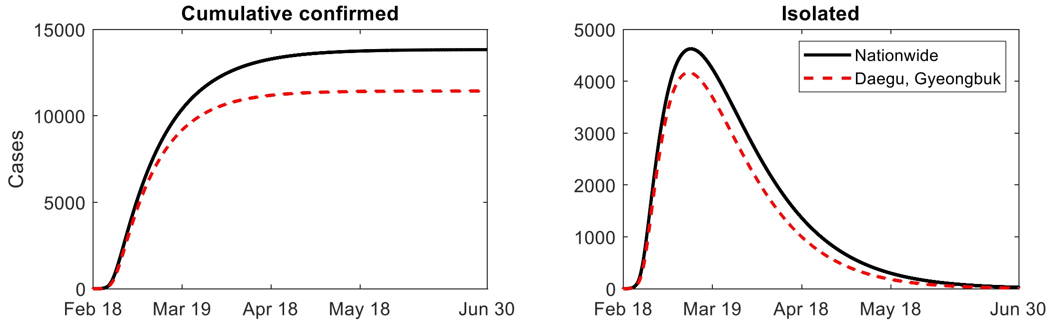

Figure 4의 왼쪽 그래프는 시간 별 누적 확진자수를 나타내고 오른쪽 그림은 시간 별 격리환자수를 나타낸다. 검은색 실선은 전국, 빨간색 파선은 대구경북지역 예측치를 나타낸다.

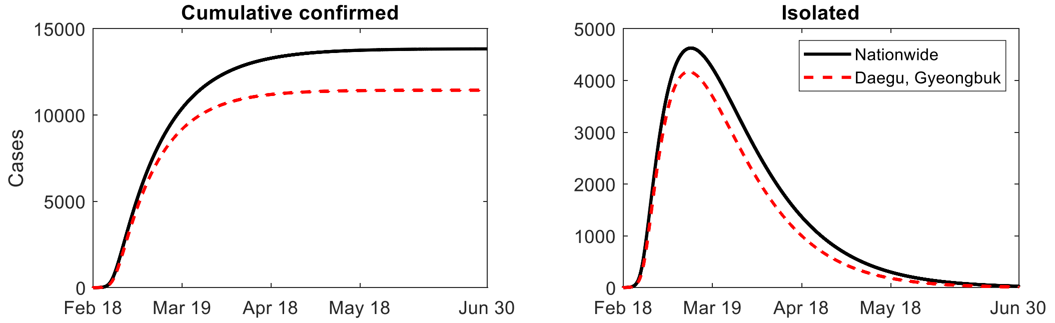

Figure 4 Model prediction using estimated parameters

모델에 따르면 전국적으로 약 13,800명이 발생할 것으로 예측되며 마지막 환자 발생일은 6월 14일이고, 대구·경북지역의 경우 5월 27일까지 약 11,400명의 환자가 발생할 것으로 예측된다. 유행 종료시점에서 대구·경북지역 환자의 비율은 82.70%이다. 10만명당 누적 확진자수는 전국 26.49명, 대구·경북지역 224.64으로 계산된다. 격리환자의 경우 전국과 대구·경북지역 모두 3월 11일에 최고 격리환자수를 지나 감소 추세로 전환될 것으로 예측된다. 모델을 통해 산출된 최고 격리환자수의 경우 전국적으로 4,625명이 예측되며 그 중 대구·경북지역은 4,167명이다.

Table 2 Model prediction

Region	Maximum isolated individuals	Date of maximum isolated individuals	Final size of epidemic	Final incidence rate per 100,000	The end of outbreak
**Nationwide**	4,625	March 11, 2020	13,830*	26.49	June 14, 2020
**Daegu, Gyeongbuk**	4,167	March 11, 2020	11,438*	224.64	May 27, 2020

* Except 30 cases before February 18, 2020

Discussion

대구·경북지역 환자들과 전국 환자들의 증상발현부터 격리까지의 기간이 같다고 가정했을 때, 전체 인구를 고려한 감염전파율(β/N)을 살펴보면 전국의 경우 1명의 감염환자가 1.3616e-7명을 감염시킨 것 반해 대구·경북지역의 경우 1.2145e-6명으로 대구·경북지역이 8.9배 이상 높게 추정된다.대구·경북지역 행동변화 전파율도 전국 평균보다 낮게 추정되었다.

유행 초반기의 경우 증상발현부터 격리까지의 기간이 5일 이상 소요되었지만 최근 질병관리본부의 적극적인 밀접접촉자 수색 및 검사로 대부분의 환자들이 증상발현 1-2일 내에 확진, 격리된다. 하지만 여전히 증상발현 후 5일 이상 지역사회에 감염전파를 일으킨 사람들이 보고되고 있어, 본 모델에서는 증상발현부터 격리까지의 기간을 평균 4일로 정하였다. 행동 변화 그룹의 전파감소율이 모델 결과에 많은 영향을 미친다. 하지만 현재 시행되고 있는 사회적 거리두기로 인한 전파감소율 연구가 진행된 적이 없기 때문에 본 연구에서는 재택근무 인원과 개학 연기를 고려하여 전파율이 약 1/50 이상 감소할 것으로 판단하였다. 본 모델에서 모수 δ가 감소하면 총 확진자수는 감소하고 유행기간은 짧아진다.

본 연구에서는 감수성 인구의 행동변화가 일어난 후의 감수성 그룹은 낮은 감염전파율을 유지한다고 가정하였다. 하지만 3월 24일 이후 개학으로 인해 접촉자수 증가, 환자수 감소에 따른 행동변화 그룹의 유행 인지 둔감화는 미 확진 감염자로 인한 또 다른 집단 발생을 야기시킬 가능성이 있다.

본 모델에서는 환자가 확진과 동시에 격리가 되어 더 이상 바이러스를 전파하지 않는다고 가정하였으므로 회복기간과 사망률은 총 환자수에 영향을 미치지 않으며 격리 환자수 및 필요 병상 개수 산정에만 영향을 미친다. 회복기간이 길어질수록 일일 격리 환자수는 증가하고 사망률이 증가할수록 일일 격리 환자수는 감소한다.

Conclusion

코로나바이러스감염증-19의 경우 특정지역에서의 대규모 집단발생이 특징적이다. 지역내에서의 대규모 유행은 해당지역 의료시설의 수용가능인원을 초과하여 확진 환자들이 의료시설이 수용되지 못하고 자가격리 중에 또 다른 환자를 발생시켰다. 모델링을 통한 시간별 격리환자수 예측을 통해 필요 병상 개수를 미리 파악하고 대비대응책을 수립할 필요가 있다.

우리나라의 경우 대구·경북 지역에서 대규모 유행이 일어났지만, 수리모델링 결과에서 보여주듯이 대구·경북 지역을 제외한 전국 지역에서도 약 1,000명 가까운 환자가 발생할 수 있음을 확인할 수 있다. 정부의 적극적인 중재 정책으로 초중고 개학연기, 대규모 집회자제 등 강력한 비약물적 제재가 동반되고 있다. 유행이 6월까지 지속될 것으로 예측됨을 고려하였을 때, 현재 시행되고 있는 비약물적 중재가 지속적으로 시행되기는 어려울 것이라고 판단된다. 따라서 개학 이후에도 마스크 착용, 손 씻기 등 개인위생을 더욱 더 강화하고 의심증상 발현 시 밀접접촉 차단 및 빠른 의료기관 내원을 권장, 그리고 증상발현부터 격리기간의 단축 등의 적극적 중재전략이 필수적이다.

REFERENCES

1. World Health Organization. Statement on the second meeting of the International Health Regulations (2005) Emergency Committee regarding the outbreak of novel coronavirus (2019-nCoV); 2020 Jan 30 [cited 2020 Mar 10]. Available from: https://www.who.int/news-room/detail/30-01-2020-statement-on-the-second-meeting-of-the-international-health-regulations-(2005)-emergency-committee-regarding-the-outbreak-of-novel-coronavirus-(2019-ncov).

2. World Health Organization. Coronavirus disease 2019 (COVID-19): situation report – 48; 2020 Mar 8 [cited 2020 Mar 10]. Available from: https://www.who.int/docs/default-source/coronaviruse/situation-reports/20200308-sitrep-48-covid-19.pdf?sfvrsn=16f7ccef_4.

3. Korea Centers for Disease Control and Prevention. Brief report of government meeting for

COVID-19 spread in Korea [cited 2020 Apr 14]. Available from: http://ncov.mohw.go.kr/upload/viewer/skin/doc.html?fn=1582449957863_20200223182559.hwp&rs=/upload/viewer/result/202004/ (Korean, author's translation).

4. Korea Centers for Disease Control and Prevention. Korean situation report of COVID-19; 2020 Mar 9 [cited 2020 Apr 14]. Available from: https://is.cdc.go.kr/upload_comm/syview/doc.html?fn=158371642528200.pdf&rs=/upload_comm/docu/0015/ (Korean, author's translation).

5. Korea Centers for Disease Control and Prevention (KCDC). KCDC press [cited 2020 Apr 14]. Available from: https://www.cdc.go.kr/board/board.es?mid=a20501000000&bid=0015 (Korean, author's translation).

6. Korean Statistical Information Service. Resident registration population by administrative district and age [cited 2020 Apr 14]. Available from: http://kosis.kr/statHtml/statHtml.do?orgId=101&tblId=DT_1B04006&conn_path=I2 (Korean, author's translation).

7. Perra N, Balcan D, Goncalves B, Vespignani A. Towards a characterization of behavior-disease models. PLoS One 2011;6:e23084.

8. Korea Centers for Disease Control and Prevention. Korean situation report of COVID-19; 2020 Feb 16 [cited 2020 Apr 14]. Available from: https://is.cdc.go.kr/upload_comm/syview/doc.html?fn=158184011101700.hwp&rs=/upload_comm/docu/0015/ (Korean, author's translation).

9. Ki M; Task Force for 2019-nCoV. Epidemiologic characteristics of early cases with 2019 novel coronavirus (2019-nCoV) disease in Korea. Epidemiol Health 2020;42:e2020007.
